# Chronic Spinal Pain: Pathophysiology, Diagnosis, and Treatment

**DOI:** 10.1155/2019/1729059

**Published:** 2019-08-15

**Authors:** Baogan Peng, Nikolai Bogduk, Michael J. DePalma, Ke Ma

**Affiliations:** ^1^Department of Spinal Surgery, The Third Medical Center of PLA General Hospital, Beijing 100039, China; ^2^Faculty of Medicine and Health Sciences, The University of Newcastle, Newcastle, Australia; ^3^Interventional Spine and Musculoskeletal Care, Virginia iSpine Physicians, PC Virginia Spine Research Institute, Inc., Richmond, VA, USA; ^4^Pain Department, Xinhua Hospital, Shanghai Jiao Tong University, Shanghai, China

Chronic spinal pain is one of the most leading causes of disability among adults worldwide. It covers chronic neck, thoracic, and low back pain. Despite the frequency of this presenting complaint, a clear understanding of its etiologies is often elusive. Any innervated spinal structures, such as muscles, synovial joints, intervertebral discs, dura mater, and ligaments, may cause pain theoretically. Because the diagnosis of chronic spinal pain is difficult, its treatment is usually nonspecific. This special issue seeks to cover chronic spinal pain-related basic and clinical studies.

Pulsed radiofrequency (PRF) is effective in the treatment of spinal pain and neuropathic pain. The research of X. R. Xu et al. showed that microglial BDNF, PI3K, and p-ERK in spinal cord are suppressed by the treatment of PRF on DRG to ease SNI-induced neuropathic pain in rats, which provided a theoretical basis for the clinical spinal pain management. J. N. Wang et al. showed that intrathecal administration of low-concentration oxygen/ozone alleviated mechanical allodynia and attenuated radiculitis, likely by a PDE2A-cGMP/cAMP-NF-kappa B/p65 signaling pathway in chronic radiculitis rats. This study provided a new theoretical basis for the treatment of spinal radiculitis pain. The study from J. Yang et al. founded that the mice SMIR model presented a persistent pain syndrome, including evoked pain and spontaneous pain. Clonidine and gabapentin can relieve mechanical hypersensitivity dose-dependent simultaneously. However, clonidine but not gabapentin can alleviate the spontaneous pain of SMIR in the mice model. It may provide a new choice in the prevention and treatment of postoperational spinal-related pain.

Spinal pain is a kind of neuropathic pain. S. F. Husain et al. suggested that matrix metalloproteinases-12 (MMP-12) is a potential biomarker of neuropathic pain. Its detection in vivo, using IONP enhanced MRI, may be further developed as a tool for neuropathic pain diagnosis and management. Y. F. Chen et al.'s article showed that the existence of MCs (Modic changes) affects the outcomes of nonsurgical treatment in patients with LBP. However, symptoms can be improved after an additional round of treatment for Modic type I changes, while this is not confirmed for Modic type II changes.

How to diagnose and treat spinal pain effectively and safely is the key part of this special issue. The special issue accepted three consensuses from the CASP (Chinese Association for the Study of Pain): Cervicogenic headache consensus emphasized the early diagnosis of cervical headache and the appropriate time for minimally invasive interventional therapy. Osteoarthritis consensus explained the whole process management of prevention, rehabilitation, and pain diagnosis and treatment of osteoarthritis. Nonspecific low back pain consensus showed the importance of correct diagnosis and effective treatment of nonspecific low back pain. Three consensuses will provide a high-quality reference for the formulation of pain diagnosis and treatment strategies in different disciplines. P. Rigoard et al.'s study showed that the utilization of a multidisciplinary approach is emphasized to ensure that care is provided in a uniform manner to reduce variation in practice and improve patient outcomes. B. B. Wu et al.'s paper showed that the safety and effectiveness of transforaminal percutaneous endoscopic lumbar discectomy with foraminoplasty (TF PELF) are comparable to TF percutaneous endoscopic lumbar discectomy (PELD) for LDH patients. The study from W. S. Yuan et al. suggested that there is a difference between scoliosis and nonscoliosis in the treatment of nonspecific low back pain. L. Shen et al. pointed out that the treatment of chronic pain varies from region to region in the same country.

